# The Role of Conspiracist Ideation and Worldviews in Predicting Rejection of Science

**DOI:** 10.1371/journal.pone.0075637

**Published:** 2013-10-02

**Authors:** Stephan Lewandowsky, Gilles E. Gignac, Klaus Oberauer

**Affiliations:** 1 School of Psychology, University of Western Australia, Crawley, Australia; 2 Department of Experimental Psychology, University of Bristol, Bristol, United Kingdom; 3 Department of Psychology, University of Zurich, Zurich, Switzerland; The University of New South Wales, Australia

## Abstract

**Background:**

Among American Conservatives, but not Liberals, trust in science has been declining since the 1970's. Climate science has become particularly polarized, with Conservatives being more likely than Liberals to reject the notion that greenhouse gas emissions are warming the globe. Conversely, opposition to genetically-modified (GM) foods and vaccinations is often ascribed to the political Left although reliable data are lacking. There are also growing indications that rejection of science is suffused by conspiracist ideation, that is the general tendency to endorse conspiracy theories including the specific beliefs that inconvenient scientific findings constitute a “hoax.”

**Methodology/Principal findings:**

We conducted a propensity weighted internet-panel survey of the U.S. population and show that conservatism and free-market worldview strongly predict rejection of climate science, in contrast to their weaker and opposing effects on acceptance of vaccinations. The two worldview variables do not predict opposition to GM. Conspiracist ideation, by contrast, predicts rejection of all three scientific propositions, albeit to greatly varying extents. Greater endorsement of a diverse set of conspiracy theories predicts opposition to GM foods, vaccinations, and climate science.

**Conclusions:**

Free-market worldviews are an important predictor of the rejection of scientific findings that have potential regulatory implications, such as climate science, but not necessarily of other scientific issues. Conspiracist ideation, by contrast, is associated with the rejection of all scientific propositions tested. We highlight the manifold cognitive reasons why conspiracist ideation would stand in opposition to the scientific method. The involvement of conspiracist ideation in the rejection of science has implications for science communicators.

## Introduction

The U.S. public has become increasingly polarized in their attitudes towards science. Since the 1970's, Conservatives—unlike Liberals or Moderates—have become increasingly skeptical and distrustful of science [1]. Polarization is particularly pronounced with respect to climate change: People who embrace a laissez-faire vision of the free market are less likely to accept that anthropogenic greenhouse gas emissions are warming the planet than people with an egalitarian-communitarian outlook [2]–[7]. Although the crucial role of cultural worldviews in determining beliefs about climate science is now well established, at least two important questions remain unanswered.

First, it is unknown how worldviews shape people's opinions about other controversial scientific issues, such as genetically-modified (GM) foods and childhood vaccinations, both of which have attracted considerable opposition. A better understanding of the role of worldview vis-á-vis those issues is important not only in its own right but also because it can triangulate the reasons *why* climate science has become so ideologically disputed. For example, if fear of government regulation of businesses were the sole factor underlying Conservatives' opposition to climate science [8], then one would expect them to embrace GM foods, like other new technologies [9], because of the associated business opportunities. If Conservatives were found to oppose GM foods, by contrast, this would point towards a more general opposition to science that transcends pragmatic considerations. Although media reports have implicated the political Left in the opposition to GM foods [10], [11], European surveys have variously associated GM-food rejection with the extreme political Right [12] as well as the political Left [13]. We are not aware of any equivalent peer-reviewed research in the U.S. A similar ambiguity arises with respect to vaccinations. Media reports have ascribed an anti-vaccine stance to the political Left [14], largely based on the political leanings of spokespersons. By contrast, research has linked opposition to mandatory human-papillomavirus (HPV) immunizations against cervical cancer to free-market and individualistic worldviews [15], perhaps reflecting fears of government intrusion into parental sovereignty. Likewise, social conservatives have taken a contrarian stance because HPV is transmitted primarily through sexual contact, thereby associating vaccinations with potential promiscuity [16]. To resolve these ambiguities, we examined the role of worldviews in determining the American public's attitudes towards GM foods and vaccinations using established measures of worldviews in a representative survey.

Second, a striking feature of the opposition to climate science is that worldview-driven polarization often increases with greater levels of education [3] and greater science literacy [17], suggesting that the opposition reflects a cognitive *style* rather than a deficit of knowledge or ability. One cognitive style that has been repeatedly implicated in science denial is conspiratorial thinking [18]–[21], also known as conspiracist ideation. Denial of the link between HIV and AIDS frequently involves conspiracist hypotheses, for example that AIDS was created by the U.S. Government [22]–[24]. Popular books critical of climate science routinely refer to global warming as a “conspiracy” or “hoax” [25], and conspiracist themes have been identified in climate media coverage [26] and in people's affective imagery evoked by climate change [21]. Among visitors to climate blogs, the tendency to endorse conspiracy theories has been shown to be correlated with the rejection of climate science as well as the rejection of other scientific propositions [27]. Likewise, analyses of YouTube videos critical of HPV vaccinations [28] and anti-vaccination blogs [29] have revealed widespread conspiratorial content.

The prominence of conspiracist ideation in science rejection is not unexpected in light of its cognitive attributes: For example, if a scientific consensus cannot be accepted as the result of researchers converging independently on the same evidence-based view, then the belief in a scientific conspiracy can provide an alternative explanation for the consensus [18], [20], [21]. Moreover, because conspiracist ideation need not conform to the criteria of consistency and coherence that characterize scientific reasoning [30], its explanatory reach is necessarily greater than that of competing (scientific) theories [31]. Conspiracist ideation is also typically immune to falsification because contradictory evidence (e.g., climate scientists being exonerated of accusations) can be accommodated by broadening the scope of the conspiracy (exonerations are a whitewash), often with considerable creativity [32]. Those cognitive attributes render conspiracist ideation ideally suited for the ongoing rejection of scientific evidence. Notwithstanding the growing prominence of conspiracist ideation in science denial, broad-based empirical data on its role are lacking. Our survey therefore also probed conspiracist ideation.

We related three potential predictors—endorsement of the free market, conservatism-liberalism, and conspiracist ideation—to people's attitudes concerning three contentious scientific issues—climate science, vaccinations, and GM foods. Each construct was measured by a number of diverse items, thereby assaying people's general attitudes (e.g., towards vaccinations and GM foods generally) rather than specific opinions (e.g., concerning HPV immunization or “Roundup-ready” maize). All items other than those targeting GM foods and vaccinations were used in previous research (see *Materials* for details) and have a track record of construct validity.

The conspiracist ideation items were sub-divided into those that probed general conspiracies (e.g., “Princess Diana's death was an organized assassination”) and others that probed specific scientific conspiracies (“The alleged link between second-hand tobacco smoke and ill health reflects bogus and corrupt science”). The latter “convenience” theories illustrate the extent to which rejection of a scientific proposition entails the belief that the relevant evidence is the result of a conspiracy among scientists. The former, general conspiracy items tap people's overall propensity for conspiracist ideation and show whether this general cognitive style is associated with the rejection of scientific propositions. Table 1 provides a verbatim list of all items together with brief labels for the items (e.g., *CYAIDS* for “U.S. agencies intentionally created the AIDS epidemic and administered it to Black and gay men in the 1970s”) that are used for presentation of the results.

**Table 1:** Questionnaire items used in the survey and their short names.

Item name	Item (R = reverse scored)
1. Climate science	
*CNatFluct*	I believe that the climate is always changing and what we are currently observing is just natural fluctuation. (R)
*CdueGHG*	I believe that most of the warming over the last 50 years is due to the increase in greenhouse gas concentrations.
*CseriousDamage*	I believe that the burning of fossil fuels over the last 50 years has caused serious damage to the planet's climate.
*CO2causesCC*	Human CO2 emissions cause climate change.
HumansInsign	Humans are too insignificant to have an appreciable impact on global temperature. (R)

2. GM Foods	
*GMimportant*	I believe that genetic modification is an important and viable contribution to help feed the world's rapidly growing population.
*GMdamageEnv*	I believe genetically engineered foods have already damaged the environment. (R)
*GMtested*	The consequences of genetic modification have been tested exhaustively in the lab, and only foods that have been found safe will be made available to the public.
*GMdangerous*	I believe that because there are so many unknowns, that it is dangerous to manipulate the natural genetic material of foods. (R)
*GMsafe*	Genetic modification of foods is a safe and reliable technology.

3. Vaccinations	
*VaxSafe*	I believe that vaccines are a safe and reliable way to help avert the spread of preventable diseases.
*VaxNegSide*	I believe that vaccines have negative side effects that outweigh the benefits of vaccination for children. (R)
*VaxTested*	Vaccines are thoroughly tested in the laboratory and wouldn€t be made available to the public unless it was known that they are safe.
*VaxRisky*	The risk of vaccinations to maim and kill children outweighs their health benefits. (R)
*VaxContribHealth*	Vaccinations are one of the most significant contributions to public health.


4. Conservatism – Liberalism	
*PLiberal*	I am politically more liberal than conservative. (R)
*PRepub*	In any election, given a choice between a Republican and a Democratic candidate, I will select the Republican over the Democrat.
*PCommunismFailed*	Communism has been proven to be a failed political ideology.
*PNeverConserv*	I cannot see myself ever voting to elect conservative candidates. (R)
*PMediaLeft*	The major national media are too left-wing for my taste.
*PSocialismOK*	Socialism has many advantages over capitalism. (R)
*PLeft*	On balance, I lean politically more to the left than to the right. (R)

5. Free market	
*FMUnresBest*	An economic system based on free markets unrestrained by government interference automatically works best to meet human needs.
*FMLimitSocial*	The free market system may be efficient for resource allocation but it is limited in its capacity to promote social justice. (R)
*FMMoreImp*	The preservation of the free market system is more important than localized environmental concerns.
*FMThreatEnv*	Free and unregulated markets pose important threats to sustainable development. (R)
*FMUnsustain*	The free market system is likely to promote unsustainable consumption. (R)

6. Conspiracist ideation	
*CYNewWorldOrder*	A powerful and secretive group known as the New World Order are planning to eventually rule the world through an autonomous world government which would replace sovereign governments.
*CYMLK*	The assassination of Martin Luther King Jr. was the result of an organized conspiracy by U.S. government agencies such as the CIA and FBI.
*CYMoon*	The Apollo moon landings never happened and were staged in a Hollywood film studio.
*CYJFK*	The assassination of John F. Kennedy was not committed by the lone gunman Lee Harvey Oswald but was rather a detailed organized conspiracy to kill the President.
*CY911*	The U.S. government allowed the 9–11 attacks to take place so that it would have an excuse to achieve foreign (e.g., wars in Afghanistan and Iraq) and domestic (e.g., attacks on civil liberties) goals that had been determined prior to the attacks.
*CYDiana*	Princess Diana's death was not an accident but rather an organised assassination by members of the British royal family who disliked her.

7. Convenience conspiracy theories	
*CYClimChange*	The claim that the climate is changing due to emissions from fossil fuels is a hoax perpetrated by corrupt scientists who wish to spend more taxpayer money on climate research.
*CYAIDS*	U.S. agencies intentionally created the AIDS epidemic and administered it to Black and gay men in the 1970s.
*CYTobacco*	The alleged link between second-hand tobacco smoke and ill health is based on bogus science and is an attempt by a corrupt cartel of medical researchers to replace rational science with dogma.

8. Other sciences	
*CauseHIV*	The HIV virus causes AIDS.
*CauseSmoke*	Smoking causes lung cancer.
*CauseLead*	Lead in drinking water poses a serious long-term health risk.

## Results

### Summary of the sample

**Figure 1:**
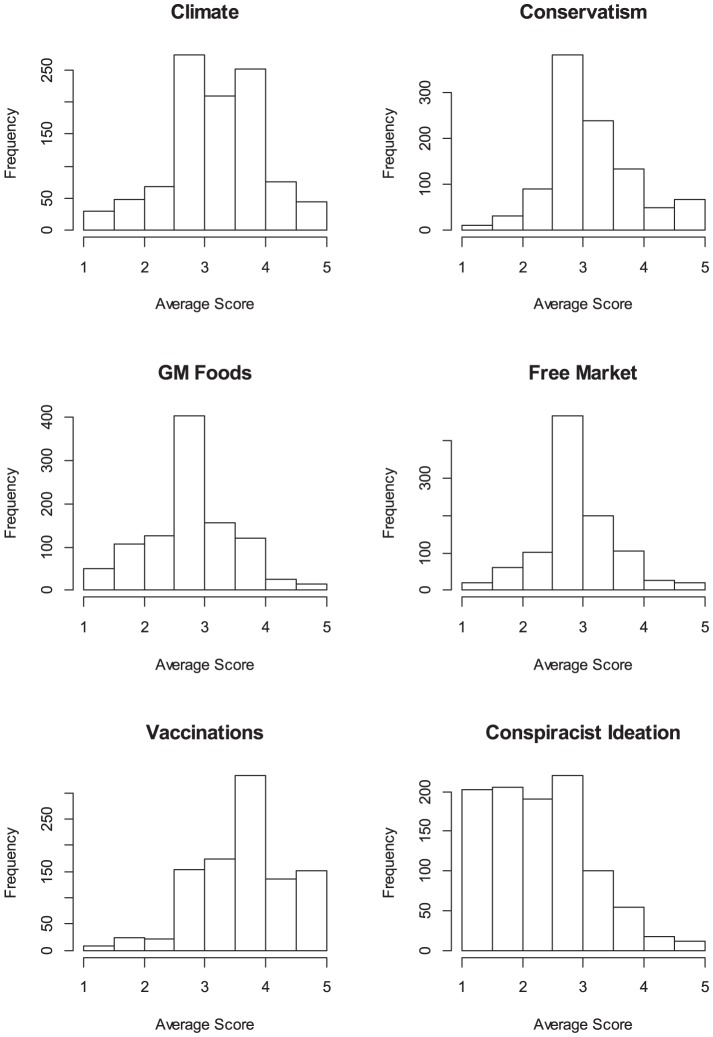
Frequency distributions of the single-indicator composite scores for all 6 constructs. Each histogram shows the distribution across subjects of the single-indicator scores. Each variable represents the average responses across the constituent items on the 5-point from ‘Strongly Disagree’ (1) to ‘Strongly Agree’ (5), with ‘Neutral’ representing the midpoint.

Table 2 summarizes the 6 constructs that entered into our main latent-variable analysis (see *Materials and Methods* section for details of their construction). Figure 1 shows the underlying distributions of the single-indicator composite scores. The apparent departure from normality of the conspiracist ideation indicator is considered during the SEM modeling via boot-strapping of confidence intervals for the parameter estimates.

**Table 2:** Descriptive Statistics Associated with Indicator Composite Scores.

Variable	*M*	Skew	Kurtosis	Min	Max	*SD*	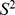	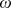	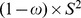
Climate	3.24	−0.38	0.45	1	5	0.77	0.6	0.728	0.163
GM Food	2.84	−0.10	0.31	1	5	0.73	0.54	0.773	0.121
Vaccines	3.70	−0.51	0.68	1	5	0.74	0.54	0.707	0.160
Conservatism	3.18	0.48	0.55	1.14	5	0.70	0.5	0.659	0.169
Free Market	2.98	0.13	1.38	1	5	0.64	0.41	0.60	0.164
Conspiracist	2.37	0.37	−0.22	1	5	0.87	0.75	0.844	0.117

*Note.* Composite scores are means across items on the 5-point scale. 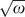 corresponds to the loading of a single-indicator manifest variable on its factor. 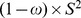 refers to the error variance of each single-indicator latent variable. For comparison, the corresponding Cronbach's 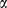
's for the Climate, GM Food, Vaccines, Conservatism, Free Market, and Conspiracist composite scores were estimated at .781, .807, .778, .774, .667, and .842, respectively.

Table 3 provides a break-down of the distribution of responses for all of the individual items.

**Table 3:** Distribution of responses to survey items.

Item Name	Strongly Disagree		Disagree		Neutral		Agree		Strongly Agree	
*CNatFluct*	152	(15.2)	353	(35.3)	235	(23.5)	218	(21.8)	43	(4.3)
*CdueGHG*	47	(4.7)	129	(12.9)	359	(35.9)	355	(35.5)	111	(11.1)
*CseriousDamage*	46	(4.6)	110	(11)	279	(27.9)	401	(40.1)	165	(16.5)
*CO2causesCC*	57	(5.7)	154	(15.4)	390	(39)	315	(31.5)	85	(8.5)
*HumansInsign*	64	(6.4)	151	(15.1)	241	(24.1)	352	(35.2)	193	(19.3)
*GMimportant*	74	(7.4)	201	(20.1)	403	(40.3)	254	(25.4)	69	(6.9)
*GMdamageEnv*	68	(6.8)	240	(24)	454	(45.4)	190	(19)	49	(4.9)
*GMtested*	70	(7)	201	(20.1)	434	(43.4)	243	(24.3)	53	(5.3)
*GMdangerous*	142	(14.2)	380	(38)	323	(32.3)	130	(13)	26	(2.6)
*GMsafe*	113	(11.3)	266	(26.6)	423	(42.3)	165	(16.5)	34	(3.4)
*VaxSafe*	26	(2.6)	48	(4.8)	163	(16.3)	469	(46.9)	295	(29.5)
*VaxNegSide*	54	(5.4)	120	(12)	252	(25.2)	379	(37.9)	196	(19.6)
*VaxTested*	32	(3.2)	89	(8.9)	278	(27.8)	455	(45.5)	147	(14.7)
*VaxRisky*	50	(5)	146	(14.6)	295	(29.5)	309	(30.9)	201	(20.1)
*VaxContribHealth*	23	(2.3)	56	(5.6)	178	(17.8)	454	(45.4)	290	(29)
*PLiberal*	107	(10.7)	230	(23)	332	(33.2)	205	(20.5)	127	(12.7)
*PRepub*	178	(17.8)	217	(21.7)	341	(34.1)	162	(16.2)	103	(10.3)
*PCommunismFailed*	18	(1.8)	72	(7.2)	307	(30.7)	354	(35.4)	250	(25)
*PNeverConserv*	70	(7)	150	(15)	379	(37.9)	236	(23.6)	166	(16.6)
*PMediaLeft*	65	(6.5)	186	(18.6)	474	(47.4)	181	(18.1)	95	(9.5)
*PSocialismOK*	46	(4.6)	189	(18.9)	416	(41.6)	207	(20.7)	143	(14.3)
*PLeft*	65	(6.5)	186	(18.6)	474	(47.4)	181	(18.1)	95	(9.5)
*FMUnresBest*	67	(6.7)	176	(17.6)	394	(39.4)	278	(27.8)	86	(8.6)
*FMLimitSocial*	76	(7.6)	294	(29.4)	455	(45.5)	138	(13.8)	38	(3.8)
*FMMoreImp*	62	(6.2)	239	(23.9)	420	(42)	218	(21.8)	62	(6.2)
*FMThreatEnv*	64	(6.4)	270	(27)	391	(39.1)	200	(20)	76	(7.6)
*FMUnsustain*	57	(5.7)	198	(19.8)	467	(46.7)	208	(20.8)	71	(7.1)
*CYNewWorldOrder*	240	(24)	259	(25.9)	318	(31.8)	120	(12)	64	(6.4)
*CYMLK*	276	(27.6)	279	(27.9)	294	(29.4)	104	(10.4)	48	(4.8)
*CYMoon*	492	(49.2)	267	(26.7)	165	(16.5)	51	(5.1)	26	(2.6)
*CYJFK*	172	(17.2)	192	(19.2)	311	(31.1)	224	(22.4)	102	(10.2)
*CY911*	401	(40.1)	259	(25.9)	195	(19.5)	87	(8.7)	59	(5.9)
*CYDiana*	273	(27.3)	260	(26)	290	(29)	118	(11.8)	60	(6)
*CYClimChange*	257	(25.7)	304	(30.4)	240	(24)	127	(12.7)	73	(7.3)
*CYAIDS*	447	(44.7)	258	(25.8)	192	(19.2)	65	(6.5)	39	(3.9)
*CYTobacco*	343	(34.3)	298	(29.8)	213	(21.3)	103	(10.3)	44	(4.4)
*CauseHIV*	10	(1)	43	(4.3)	122	(12.2)	378	(37.8)	448	(44.8)
*CauseSmoking*	6	(0.6)	23	(2.3)	99	(9.9)	352	(35.2)	521	(52)
*CauseLead*	152	(15.2)	353	(35.3)	235	(23.5)	218	(21.8)	43	(4.3)

*Note*. Table entries are numbers of responses (and percentages). See Table 1 for wording of items.

### Exploration of specific conspiracy theories relating to scientific propositions

The convenience conspiracies were endorsed (i.e., ratings above “Neutral”) by 200, 147, and 104 respondents (out of 1001), for Climate-Change-Hoax (*CYClimChange*), Tobacco-Lung Cancer-Hoax (*CYTobacco*), and HIV-AIDS-Conspiracy (*CYAIDS*), respectively.

The bivariate correlations between each convenience theory and the corresponding item(s) querying the scientific proposition were 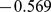 for *CYClimChange* (correlated with the average response to all 5 climate items), 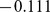 for *CYAIDS* 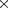 *CauseHIV*, and 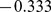 for *CYTobacco* 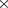 *CauseSmoking*, respectively (all 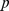
's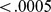). The correlations confirm that rejection of scientific propositions is often accompanied by endorsement of scientific conspiracies pertinent to the proposition being rejected.

### Modeling of science acceptance

**Figure 2:**
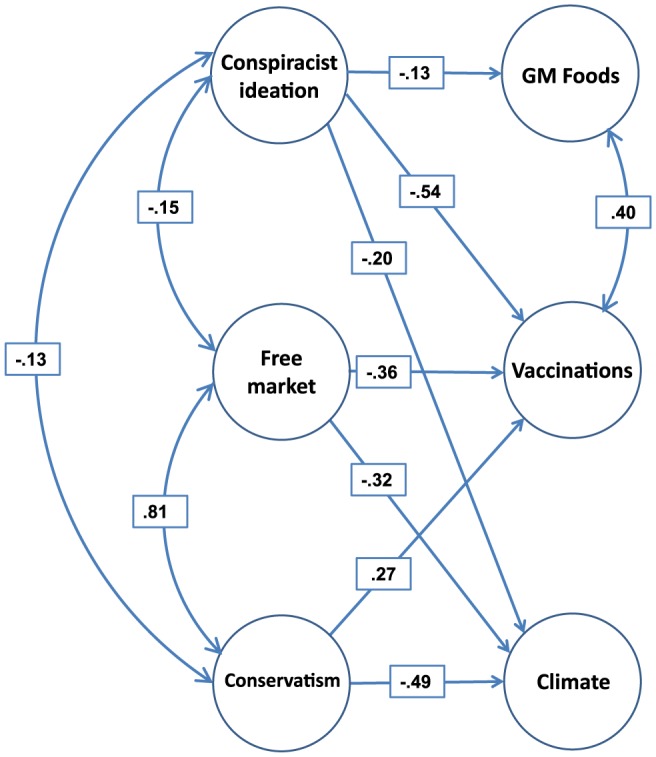
Structural Equation Model summarizing the data (Model 1). All links and correlations shown are standardized and significant; all 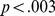 except the link between Conservatism and Vaccinations; , 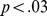. Manifest variables and their loadings, and disturbances on endogenous factors, are not shown. Links between latent variables that are not shown are constrained to zero. Loadings and variances of single-indicator manifest variables are not shown and are reported in Table 2.

Structural equation modeling (SEM; see *Materials and Methods* section) examined the relationships between the constructs of greatest interest, namely the worldview latent variables, general conspiracist ideation, and climate change, vaccinations, and GM foods. Figure 2 shows the overall SEM (Model 1) which fit very well (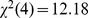, 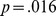, CFI 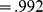, TLI = 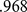, RMSEA = 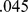; 90% CI: 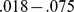, SRMR = 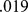, AIC = 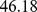) based on conventional standards (CFI and TLI 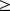.95 and RMSEA and SRMR 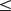.06; [33]).

Not unexpectedly, free-market endorsement and conservatism together strongly predicted rejection of climate science, with standardized weights of 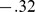 and 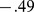, respectively. Acceptance of GM foods, by contrast, was not associated with people's worldviews (weights set to zero without significant loss of fit; 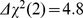, 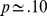). The relationship between worldviews and attitudes towards vaccinations was more complex, with free-market endorsement predicting rejection and conservatism predicting acceptance of vaccinations, respectively.

Table 4 shows that the bivariate correlations between each of the worldview predictors (free market and conservatism latent variables) and the acceptance of vaccinations were in opposing directions but numerically small and non-significant. The fact that those predictors carried considerable weight in the SEM model (Figure 2) therefore merits exploration. Further analysis revealed that the low bivariate correlations combined with greater weights in the SEM likely reflected a suppressor effect [34], [35]. A suppressor variable mediates the association between two other variables by suppressing criterion-irrelevant variance in the predictor. In the present instance, we find that much of the suppressor effect arises from the fact that conservatism and free-market worldview are substantially and positively correlated with each other (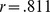) but opposingly (positively vs. negatively, respectively) associated with vaccinations. In consequence, the bivariate correlations with vaccinations are low (cf. Table 4) because, as far as each predictor on its own is concerned, the large shared variance between free-market and conservatism is “nuisance” variance—that is, variance that is irrelevant to the prediction of vaccination acceptance. Thus, when that criterion-irrelevant variance for each predictor is suppressed by the other predictor (as accomplished within the SEM model), the full strength of each predictor is revealed.

**Table 4:** Correlations among the 6 latent variables retained for the Structural Equation Modeling.

	Variable	1	2	3	4	5
1	Conspiracist ideation	–				
2	Free Market	−.142	–			
3	Conservatism	−.125	.811	–		
4	GM Food	−.129	.091	(.004)	–	
5	Vaccinations	−.521	(−.038)	(.052)	.387	–
6	Climate	−.089	−.674	−.730	(−.076)	.126

*Note*. All correlations *except* those enclosed in parentheses are significant (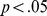). The bivariate correlations between acceptance of vaccinations and the Free Market and Conservatism predictors, respectively, were small and non-significant. See text for explanation why those predictors were nonetheless significant in the SEM model (Figure 2).

In contrast to the clearly differentiated effects of worldview, conspiracist ideation predicted rejection of all three scientific issues, albeit to quite varying extents. Figure 2 shows that the link was numerically strongest for vaccinations but also significant for GM foods and climate science. Figure 1 showed that the conspiracist-ideation single-indicator variable—unlike the others in the model—departed notably from normality. The effect of such departure from normality can be counteracted by constructing confidence intervals for the parameter estimates by bootstrapping [36]. The bootstrapped confidence intervals for the model in Figure 2 did not materially alter any of the conclusions: All regression weights reported in the figure retained their significance, with the exception of the link between conservatism and acceptance of vaccinations, which failed to reach conventional significance levels (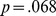). We therefore explored the role of worldview in vaccination acceptance further.

Because previous research has often relied on the free-market construct alone [4], [27], and because free-market endorsement and conservatism had opposing effects on the acceptance of vaccinations, we explored two additional models that iteratively omitted one of the worldview constructs. The additional model without conservatism (Model 2) also fit very well, 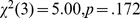, CFI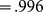, TLI = 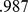, RMSEA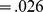; 90% CI: 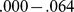, SRMR = 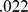, AIC = 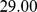, with the strength of the link between free-market endorsement and rejection of climate science rising to 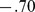. The link between free-market endorsement and rejection of vaccination declined in magnitude, 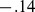, but retained its significance (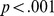). The additional model without free-market endorsement (Model 3) fit adequately, 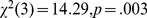, CFI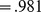, TLI = 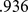, RMSEA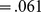; 90% CI: 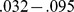, SRMR = 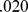, AIC = 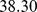, with the strength of the link between conservatism and rejection of climate science rising to 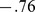. The link between conservatism and vaccination, by contrast, was no longer significant, 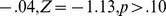. The weights involving conspiracist ideation remained virtually unchanged in these two additional models (Models 2 and 3).

## Discussion

### Worldviews and acceptance of science

We have shown that people's political orientation and worldview can present strong obstacles to acceptance of scientific evidence, albeit to widely differing extents among the issues examined. Worldviews once again constituted an overpowering barrier to acceptance of climate science. Conservatism and free-market endorsement were correlated but distinct constructs (Model 1 in Figure 2), each of which contributed a substantial share of variance to the rejection of climate science. When considered in isolation, each worldview construct on its own strongly predicted rejection of climate science (Models 2 and 3), replicating much previous research and underscoring a formidable challenge to science communicators [2]–[4], [6], [7], [27].

Recent research has shown that the role of worldview may be attenuated by underscoring the breadth of consensus among scientists: When people are informed of the pervasive consensus about the fundamentals of climate change, they become more likely to endorse the basic premise of global warming, and they attribute a larger share of the observed warming trend to human CO2 emissions [37], [38]. In one experimental study, underscoring the consensus was particularly effective for people whose worldview otherwise might have predisposed them towards rejection of climate science [38]. This experimental result meshes well with a detailed analysis of Republicans' opinions on climate change, which similarly revealed perceived consensus to be the strongest predictor of acceptance of climate science [39].

For the other two scientific propositions, the role of worldview was attenuated and more nuanced, or absent altogether. Opposition to vaccinations involved a balance between two opposing forces, namely a negative association with free-market endorsement and a compensatory positive association with conservatism. The different polarity of those associations is consonant with the notion that libertarians object to the government intrusion arising from mandatory vaccination programs [15], whereas people low on conservatism—who, by implication, are liberal or progressive—may oppose immunization because they distrust pharmaceutical companies [40]. The latter link, however, was far from overwhelming: When conservatism was considered on its own (Model 3), it was no longer associated with vaccination rejection. Conversely, free-market endorsement on its own (Model 2) predicted rejection of vaccinations, albeit more weakly than when both constructs were present (Model 1). The clear differentiation between conservatism and free-market worldviews with respect to vaccinations is notable in light of their strong bivariate correlation (cf. Table 4). Although conservatism and libertarian worldviews tend to be perceived as allied or nearly synonymous when viewed through the conventional “right vs. left” political lens, recent research has begun to differentiate libertarian worldviews from conservatism [41].

Finally, opposition to GM foods was not associated with the worldview constructs. This result is striking in light of reports in the media [10] that have linked opposition to GM foods with the political Left based on statements by political figures. Our results provide no evidence that this link holds in the American population at large. This finding is consonant with the fact that among liberals trust in science has remained high and stable since the 1970s [1]. Our data suggest that this high level of trust in science among liberals extended to GM foods. We therefore do not find much evidence for the view that the motivated rejection of scientific findings is symmetrical on both the political Left and the Right, such that liberals reject GM foods because their close association with multinational corporations challenges their values in the same way that the regulatory implications of climate science challenges conservatives [11]. Instead, our results appear more congruent with a politically asymmetric view of the role of ideology in the rejection of science. On this view, the driving psychological force that is underlying the rejection of science is “system justification” [42], [43]; that is, a person's need to perceive the current political and economic system as fair, legitimate, and stable. According to the system justification view, scientific findings are rejected by people high in system justification when the evidence challenges the status quo [42], rather than on the basis of ideology per se. Hence, because system justification tends to be greater among conservatives than liberals [42], climate science is primarily rejected by people on the political right because they tend to be particularly concerned with system justification and hence respond to the threat to the economic status quo that might arise from climate mitigation efforts. By contrast, GM foods are not rejected based on ideology because they do not imperil the economic status quo, thereby eliminating system justification as a driving variable for rejection. We add the cautionary note that although our sample was representative, it may not have included a sufficiently large number of participants at the extreme end of the ideological spectrum. It is therefore possible that small specific groups on the political left do indeed reject certain scientific findings—such as GM foods or vaccinations—as is suggested by the public rhetoric of spokespersons that are identified as “left-wing” [10], [11].

In summary, although a free-market worldview is a powerful predictor of the rejection of scientific findings that have regulatory implications such as climate science, we found its effect to be far from general: The involvement of worldview in vaccinations was arguably small, and it was entirely absent for GM foods. Nonetheless, it must be reiterated that we found limited evidence for the rejection of vaccinations based on liberal or “left-wing” political leanings: When free-market worldviews are parceled out (and only then), people on the political left were less likely to endorse childhood vaccinations than people on the political right.

### Conspiracist ideation vs. scientific cognition

Unlike worldview, conspiracist ideation predicted rejection of all scientific propositions, albeit to varying extents. Given that none of the conspiracy items had any direct bearing on the propositions under consideration (recall that “convenience” theories were not considered in the SEM), the data provide further evidence for the link between the rejection of science and a conspiratorial cognitive style in general [18]–[20], [27].

This association is arguably not coincidental and of theoretical and practical significance. We noted at the outset that conspiracist ideation can provide an alternative explanation for a pervasive scientific consensus, a role that is arguably reflected in the pairwise negative correlations between the “convenience” theories (*CYClimChange*), *CYTobacco*, and *CYAIDS*) and the corresponding scientific propositions. It must be noted that the magnitude of those correlations was quite substantial, with *CYClimChange* explaining a third of the variance in the acceptance of climate science (bivariate 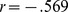; 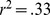). However, conspiracist ideation typically is not limited to individual theories but represents a broader cognitive style or personality attribute. People who endorse one conspiracy are known to be likely to also endorse multiple others; thus, the belief that AIDS was created by the government has often been found to be accompanied by the conviction that the FBI killed Martin Luther King or that MI6 killed Princess Diana [44], [45]. We found a similar convergence of beliefs here (cf. Table 5). In further support of a fairly general disposition towards a conspiracist style, it has been shown that endorsement of conspiracy theories is also associated with people's own willingness to engage in a conspiracy themselves when deemed necessary [46]. It is not surprising, therefore, that conspiracist ideation has been found to be associated with stable personality variables, such as a subset of the ‘Big Five’ [45] or paranoid ideation and schizotypy [47]. We nonetheless prefer to view conspiracist ideation as a cognitive style rather than a potential personality trait because if conspiracist ideation is considered at a cognitive level, its analysis can reveal why it is antithetical to scientific reasoning in several ways.

**Table 5:** Model Fit Statistics and Indices Associated with the Single-Factor Measurement Models.

Model	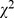	*df*	SRMR	CFI	TLI	RMSEA	AIC
Climate 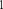	11.35	4	.016	.996	.989	.043	33.35
GM Food 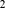	11.92	4	.016	.995	.988	.044	33.92
Vaccines 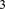	12.14	4	.016	.995	.987	.045	34.14
Conservatism 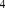	105.22	12	.039	.950	.913	.088	137.22
Free Market 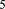	10.01	4	.020	.994	.985	.039	32.04
Conspiracist 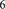	77.13	9	.032	.968	.947	.087	101.13

*Notes*. 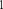 single-factor model with one correlated residual between the *CNatFluct* and *HumansInsign* items (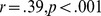; see Table 0 for explanation of variable names); 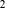 single-factor model with one correlated residual between the *GMdamageEnv* and *GMdangerous* items (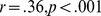); 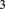 single-factor model with one correlated residual between the *VaxNegSide* and *VaxRiskyVax* items (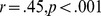); 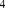 single-factor model with two correlated residuals: one between the *PRepub* and *PMediaLeft* items (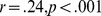), and one between the *PCommunismFailed* and *PSocialismOK* items (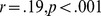); 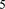 single-factor model with one correlated residual between the *FMUnresBest* and *FMMoreImp* items (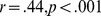); 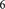 no correlated residuals were added to this single-factor model.

For example, whereas coherence is a hallmark of most scientific theories, the simultaneous belief in mutually contradictory theories—e.g., that Princess Diana was murdered but faked her own death—is a notable aspect of conspiracist ideation [30]. Accordingly, arguments against mainstream climate science are often mutually contradictory, as noted in the U.S. Environmental Protection Agency's response to comments on its endangerment finding pertaining to greenhouse gases [48]. Second, conspiracy theories rely on isolated morsels of evidence inconsistent with an official account [31] (e.g., that Timothy McVeigh fled the Oklahoma City bombing in a car without licence plates) while ignoring overwhelming evidence to the contrary (e.g., the full documented history leading up to the bombing). Accordingly, anti-vaccination activists ignore overwhelming statistical evidence while focusing on anecdotes or information that has been shown to be false [49]. Finally, in a reversal of conventional scientific reasoning, evidence *against* conspiracy theories is often construed as evidence *for* them, because the evidence is interpreted as arising from the conspiracy in question. This interpretation relies on the notion that, the stronger the evidence against a conspiracy, the more the conspirators must want people to believe their version of events [31], [32]. This self-sealing reasoning can engender baroque theories that are bedazzling in their complexity. For example, when a component of the numerous 9/11 theories became untenable—i.e., that no plane hit the Pentagon—the very fact that the no-plane theory was false was weaved into a larger 9/11 conspiracy: Specifically, the new over-arching theory held that the problem with the no-plane theory was that it was too transparently false to have been true. Thus the no-plane theory must have been a straw man initially planted by the government whose falsification was planned in order to discredit the over-arching theory that 9/11 was an inside job [32].

The resistance of conspiracist ideation to contrary evidence renders its prominence in the rejection of science particularly troubling, because providing additional scientific information may only amplify the rejection of such evidence, rather than foster its acceptance. Instead, conspiracist misconceptions of scientific issues are best met by indirect means, such as affirmation of the competence and character of proponents of conspiracy theories, or affirmation of other beliefs they hold dearly [32], [50]. Such self-affirmation is known to facilitate the dislodging of attitudes in response to information that would otherwise be considered too threatening [51]. Alternatively, efforts should be made to rebut many conspiracy theories at the same time because multiple rebuttals raise the complexity of possible conspiracist responses, thereby rendering it increasingly baroque and less believable to anyone outside a committed circle of conspiracy theorists [32].

In summary, several attributes of the cognition underlying conspiracist ideation run counter to conventional scientific thinking. The prominence of conspiracist ideation in the rejection of science should therefore not be unexpected. Knowledge of its involvement is crucial to permit scientists and communicators to respond appropriately to the rejection of evidence by segments of the public.

### Worldviews and conspiracist ideation: Birds of a feather?

What is the relationship between our two principal predictors, worldviews and conspiracist ideation? Our main SEM model showed a negative association between conspiracy theorizing and conservatism (as well as with free-market endorsement), suggesting that conspiratorial thinking is more prevalent on the political left than the right. This association is not without related precedent [45], but it is also not universal: The reverse association has also been found, whereby conspiratorial belief was linked to right-wing authoritarianism [52]. The directional lability of the association with political views may arise because some specific conspiracies are favored among the political left (e.g., that 9/11 was “an inside job”) whereas others (e.g., that President Obama was not born in the United States) are favored among the political right [53]. Depending on the balance of test items, different studies may thus yield associations with political orientation that are of different polarity. We suggest that it would be premature to tie conspiracist ideation firmly to one side of politics or the other, and that this issue awaits adjudication by further systematic research.

There is, however, an over-arching conceptual link between cultural worldviews and conspiratorial thinking: Irrespective of their statistical association (or lack thereof), they both arguably represent mechanisms of motivated reasoning. Motivated reasoning refers to the discounting of information or evidence that challenges one's prior beliefs accompanied by uncritical acceptance of anything that is attitude-consonant [54]–[56]. In the context of science denial, the present study and related precedents [2]–[7], [18]–[21], [27] have identified worldviews and conspiracist ideation as two vehicles with which such motivated reasoning is exercised. These general and robust findings help identify communicative means by which the motivated reasoning can be attenuated or circumvented [5], [32], [38], [50]. However, from a basic cognitive perspective, the next question of interest is to examine *why* people hold the worldviews they do, thereby going beyond a descriptive role of worldview to an explanatory account of the underlying cognitions and beliefs. Initial work in this direction has been promising [41], [57], [58].

## Materials and Methods

### Materials

The survey comprised 39 items, plus queries of age and gender and an attention filter question (“select Neutral”). Age turned out not to correlate with any of the indicator variables, and although gender exhibited some small associations, its inclusion as a potential mediator in the SEM model (Figure 2) did not alter the outcome (largest change in any standardized beta weight 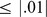.) We therefore did not consider those variables further.

All items were rated on the following 5-point rating scale: 1 =  ‘Strongly Disagree’, 2 =  ‘Disagree’, 3 =  ‘Neutral’, 4 =  ‘Agree’, and 5 =  ‘Strongly Agree.’ There were 5 items, designed for this study but derived from relevant precedents [27], [38] for each of the principal scientific issues; viz. climate, GM foods, and vaccinations. The potential predictor constructs were all based on established instruments that have been repeatedly used in previous research: free-market endorsement was measured by 5 items [4]; conservatism-liberalism by 7 items [59]; and conspiracist ideation by 6 items drawn from previous research [27], [45].

A further 3 items queried scientific propositions that were also used in previous research [27], [38]. Specifically, those items queried the link between HIV and AIDS, the link between smoking and lung cancer, and the link between lead in drinking water and adverse health effects. In addition, 3 items queried “convenience” conspiracies pertaining to specific scientific issues; viz. that climate change is a hoax, that AIDS was intentionally created by the U.S. Government, and that the link between smoking and lung cancer is based on bogus science.

Approximately half the items measuring each construct (except conspiracist ideation) were phrased such that a positive response reflected stronger endorsement, with the polarity reversed for the other half. Items were reverse-scored where necessary so that numerically greater scores represented greater endorsement. For the bipolar conservatism-liberalism construct, greater endorsement reflected greater conservatism.

Questions were presented blocked by topic area in two different orders that were randomly assigned to participants. Table 1 presents items in one order; the other order reversed the sequence.

### Participants and procedure

A sample of 1,001 U.S. residents was recruited in early June 2012 via electronic invitations by Qualtrics.com, a firm that specializes in representative internet surveys. Participants were drawn from a completely bipartisan panel of more than 5.5 million U.S. residents (as of January 2013), via propensity weighting to ensure representativeness. The panel from which participants were sampled is maintained by uSamp.com. Details about the panel and the sampling method can be found on the uSamp.com web page.

Participants were compensated by Qualtrics with cash-equivalent points. A total of 1,383 respondents entered the survey page. Of those, 74 did not enter a single response, and a further 308 either failed the attention-filter question or did not complete all items. Only participants who completed all items and passed the attention-filter question were retained for analysis. Median age of respondents retained for analysis was 43.0 (Q1: 30.0, Q3: 55.0). There were 501 male and 500 female respondents. The data set is available at the first author's webpage, www.cogsciwa.com.

### Ethics statement

The Human Research Ethics Committee of the University of Western Australia approved the procedure in conformance with the *The National Statement on Ethical Conduct in Human Research*, which is jointly promulgated by the National Health and Medical Research Council (NHMRC), the Australian Research Council (ARC), and Universities Australia (UA). The survey was prefixed by an introductory information sheet outlining the research. Participants indicated their informed consent by proceeding to the survey questions after reading this information sheet.

### Latent variable modeling

Our principal analysis used structural equation modeling (SEM). Thus, each construct of interest was represented by a latent variable estimated from the responses to the corresponding multiple items. As latent variables are free of measurement error, none of the estimated effects are attenuated due to measurement error [60]. Alternative methods of analysis, such as multiple regression based on composite scores with less than perfect reliability, yield results contaminated by measurement error which make their interpretation difficult [61].

SEM models with more than 20 indicator variables (i.e., items) are often too large to achieve adequate levels of model fit [62]. Item parceling serves to overcome this problem by averaging the item scores measuring each construct into a single-indicator variable for SEM. One criticism of item parceling is that it may obscure multi-dimensionality [63]. To preempt this criticism, we modeled each hypothesized latent variable (predictors such as conservatism and criterion variables such as acceptance of vaccinations) individually based on all of its respective items to determine their dimensionality. All six latent variables exhibited an essentially unidimensional structure (Table 5). In nearly all cases the addition of 1 correlated error term to the single-factor model was sufficient for a very respectable fit (CFI 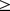.95 and SRMR 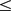.06; [33]). The only exception was the conservatism construct which included two correlated error terms.

Having confirmed essentially unidimensional structures, we modeled each construct via single-indicator latent variables [64], [65]. In single-indicator models, each latent variable is defined by one indicator consisting of an equally-weighted composite of the items within a scale (i.e., the sum or mean of the item scores). Equally weighted composites scores, rather than factor scores, were used for two principal reasons: (1) when all of the items load positively and roughly equally onto the factor, as was the case in this instance, there are no practical benefits to using factor scores [66]. In confirmation, the lowest observed correlation between factor scores and composite scores in this study was 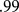 across all latent variables. (2) The estimation of internal consistency reliability of unequally weighted composite scores is more complicated and less well-established [67].

The true score variance for each latent variable is obtained by constraining the single-indicator's error variance to: 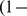
reliability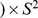, where 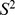 is equal to the composite score's total variance [65]. Although Cronbach's 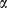 is frequently used to estimate the reliability (or true score variance) of each single-indicator variable, Cronbach's 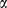 assumes essential 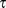-equivalence and independent error variances [68]. A more accurate estimator free of those assumptions is 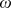 [69], [70], which was used here. As per Cronbach's 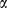, 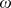 represents the ratio of true-score variance to total variance; however, it is estimated within a factor-analytic model [71]. Specifically, the individual measurement models were used to estimate the 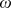 coefficients associated with each latent variable's single indicator [70] (see Table 2).

The error variances of the indicators were set to the values shown in Table 2, and the structural models for the latent variables (cf. Figure 2) were estimated using Amos 20.0. The correlation matrix for the 6 latent variables is shown in Table 4.
