# Correction: The Role of Conspiracist Ideation and Worldviews in Predicting Rejection of Science

**DOI:** 10.1371/journal.pone.0134773

**Published:** 2015-08-13

**Authors:** Stephan Lewandowsky, Gilles E. Gignac, Klaus Oberauer

This Correction is being published to provide a clarification regarding ethical approval for the inclusion of minors in this study, and to address concerns regarding the inclusion of age outliers in the dataset and some analyses that were discovered by a reader. The authors thank the reader for drawing this problem to our attention. In addition, the authors discovered a slight error in the specification of the single-indicator latent variable model for Conservatism, which necessitated an update of the fit statistics for two of the models and a slight change in the reported regression weights and correlations. A revised version of Fig 2 is included below. Note that none of the conclusions in the article are affected by these changes. The authors apologize for these errors.

**Fig 2:**
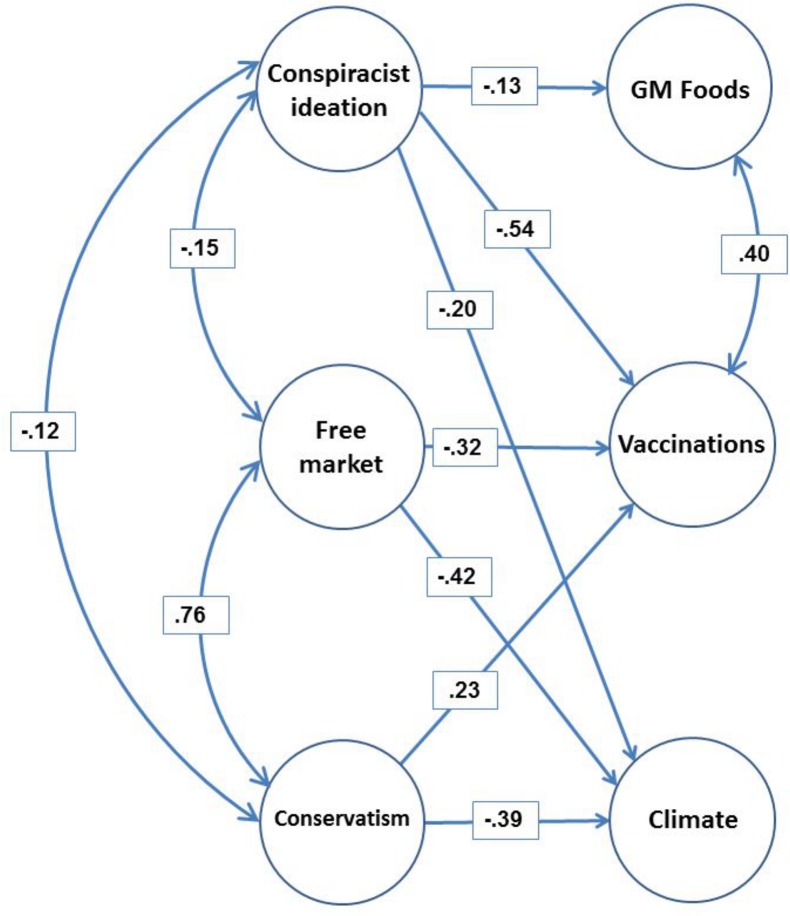
Structural Equation Model summarizing the data (Model 1). All links and correlations shown are standardized and significant; all *p* < .004 except the link between Conservatism and Vaccinations; *Z* = 2.57, *p* < .01. Manifest variables and their loadings, and disturbances on endogenous factors, are not shown. Links between latent variables that are not shown are constrained to zero. Loadings and variances of single-indicator manifest variables are not shown and are reported in Table 2.

## Ethics Statement addendum regarding inclusion of minors:

Several minors (age 14–17) were included in the data set for this study because this population contributes to public opinions on politics and scientific issues (e.g. in the classroom). This project was conducted under the guidelines of the Australian National Health and Medical Research Council (NH&MRC). According to NH&MRC there is no explicit minimum age at which people can give informed consent (as per https://www.nhmrc.gov.au/book/chapter-2-2-general-requirements-consent). What is required instead is to ascertain the young person’s competence to give informed consent. In our study, competence to give consent is evident from the fact that for a young person to be included in our study, they had to be a vetted member of a nationally representative survey panel run by uSamp.com (partner of Qualtrics.com, who collected the data). According to information received from the panel provider, they are legally empowered to empanel people as young as 13. However, young people under 15 are recruited to the panel with parental involvement. Parental consent was otherwise not required. Moreover, for survey respondents to have been included in the primary data set, they were required to answer an attention filter question correctly, further attesting to their competence to give informed consent. The UWA Human Rights Ethics Committee reviewed this issue and affirmed that “The project was undertaken in a manner that is consistent with the Australian National Statement of Ethical Conduct in Human Research (2007).”

## Correlation of age with indicator variables, and re-assessment of the structural equation model:

The dataset included two notable age outliers (reported ages 5 and 32757). As all participants must be at least 13 years old to be included in the Qualtrics panel, it was assumed that these values reflected errors of entry into the free-form age entry field on the survey. Inspection of these two records indicated nothing unusual that would suggest or mandate their exclusion. The two outliers did not affect the summary statistics for Age but did affect that variable’s correlation with other indicators, as detailed below. We examined the implications of this error, and the overall results are unaffected by removal of the outliers and inclusion of demographic covariates.

Specifically, the statement on page 9 “age turned out not to correlate with any of the indicator variables” is incorrect. It should read instead “age correlated significantly with 3 latent indicator variables (Vaccinations: .219, p < .0001; Conservatism: .169, p < .001; Conspiracist ideation: -.140, maximum likelihood p < .0001, bootstrapped p = .004), and straddled significance for a fourth (Free Market: .08, p≈.05).”

We re-analyzed the data for the structural equation model (Fig 2) excluding the two records with outlying values for age and also including Age and Gender as covariates. Table 1 shows all of the freely estimated regression path coefficients (standardized) associated with the model that includes the Age and Gender covariates (χ-square = 11.84, df = (6), p = .066, CFI = .995, TLI = .975, RMSEA = .031, SRMR = .014) together with their counterparts in the original model but with outliers removed. These changes did not notably affect any of the weights or impact the overall conclusions.

**Table 1:** 

			With age & gender as covariates, with corrected specification of conservatism	Original model (Fig 2), with corrected specification of conservatism
GM_Food	<—-	Conspiracist	-.14	-.13
Vaccines	<—-	Conspiracist	-.52	-.54
Vaccines	<—-	Freemarket	-.31	-.32
Vaccines	<—-	Conservatism	.21	.23
Climate	<—-	Conspiracist	-.20	-.20
Climate	<—-	Conservatism	-.37	-.39
Climate	<—-	Freemarket	-.43	-.42

## Revised specification of the single-indicator model for conservatism:

The single-indicator model for Conservatism was incorrectly specified (weights from two items mistakenly set to 0 rather than 1) and the correct value of ω in Table 2 is .753 (as opposed to .659), with (1-ω)×S^2^ = .124. This error does not affect any of the conclusions or alter the results substantively, but it requires correction to several weights in Fig 2 and correlations in Table 4, both of which report results for the entire sample (N = 1001). Specifically, the correlations involving Conservatism in Table 4 are -.117 with Conspiracist ideation (as opposed to -.125); .760 with Free market (as opposed to .811); .049 with Vaccinations (as opposed to .052); and -.685 with Climate (as opposed to -.730). The fit indices for Model 1 and Model 3 change slightly. Model 1: χ^2^(4) = 10.84, p = .028, CFI = .993, TLI = .974, RMSEA = .041; 90%CI: .012 - .072, SRMR = .019, AIC = 44.84. The same set of correlations and regression weights was significant as before. As before, setting the weights from the two worldview predictors to GM to zero does not significantly alter fit, Δχ^2^(2) 5.77, *p* ≈.06; bootstrapped p-values for Conservatism p = .131 and Free market p = .066 in full model. All regression weights reported in Fig 2 now retain significance after bootstrapping. Model 3: χ^2^(3) = 14.87, p = .002, CFI = .980, TLI = .933, RMSEA = .063, 90% CI: .034 - .096, SRMR = .020, AIC = 38.87, with the link between Conservatism and rejection of climate science rising to -.71. The link between conservatism and vaccination is now -.03, *Z* = -.83, *p* >.10 as before. Model 2 is unaffected. None of the weights or correlations involving conspiracist ideation are affected anywhere.
